# Convulsive Syncope Due to Complete Atrioventricular Block in a Remote Area: The Critical Role of Helicopter Emergency Medical Services

**DOI:** 10.3390/life16060895

**Published:** 2026-05-26

**Authors:** Ioana Năvălici, Mihai-Cristian Marinescu, Gabriel Ioan Prada, Cezarina Mihaela Mehedinți, Mihaela Alina Călin, Mihai Grecu, Sorina Nicoleta Munteanu, Diana Oprea, Aurelia Romila

**Affiliations:** 1 “Saint Apostle Andrew” County Emergency Clinical Hospital, 800578 Galați, Romania; cardiologmihaimarinescu@gmail.com (M.-C.M.); alina.calin@ugal.ro (M.A.C.); sorinanicoleta.munteanu@yahoo.com (S.N.M.); 2 National Institute of Gerontology and Geriatrics “Ana Aslan”, 011241 Bucharest, Romania; giprada@gmail.com; 3 “Carol Davila” University of Medicine and Pharmacy, 800201 Bucharest, Romania; 4 Faculty of Medicine and Pharmacy, “Dunărea de Jos” University of Galați, 020022 Galați, Romania; mihaela_hincu10@yahoo.com (C.M.M.); mihai.grecu@ugal.ro (M.G.); aurelia.romila@yahoo.com (A.R.); 5 Geriatric Department, Sjællands Universitetshospital Nykøbing Falster, 4800 Nykøbing Falster, Denmark; diana.v.oprea@gamil.com

**Keywords:** air medical transport, complete atrioventricular block, convulsive syncope, geriatric syncope, helicopter emergency medical services, permanent pacemaker, temporary cardiac pacing

## Abstract

**Background**: Syncope in older adults is a frequent and diagnostically challenging presentation in prehospital emergency care, particularly in geographically remote areas with limited access to advanced cardiac services. While many syncopal episodes are benign, some are caused by life-threatening conduction disorders such as complete atrioventricular block (CAVB), which may manifest with seizure-like activity due to severe cerebral hypoperfusion. **Case Presentation**: We report the case of a 70-year-old man with a history of hypertension, residing in a remote rural area, who experienced a nocturnal tonic–clonic episode followed by recurrent loss of consciousness. Due to difficult ground access, a helicopter emergency medical service (HEMS) team was deployed. On arrival, the patient was hypotensive and severely bradycardic, with recurrent syncopal episodes. Electrocardiography (ECG) confirmed CAVB with a slow escape rhythm. Temporary transvenous pacing was initiated but required progressively higher output, up to approximately 80 mV, with intermittent loss of capture, indicating a high risk of pacing failure and asystole. The patient was transported by helicopter to a tertiary interventional cardiology center, where he experienced additional syncopal episodes before undergoing emergent implantation of a permanent single-chamber ventricular pacemaker (VVI). Permanent pacing resulted in immediate hemodynamic stabilization, and no further syncopal or seizure-like episodes were observed. **Conclusions**: In geriatric patients presenting with convulsive syncope, cardiac causes such as CAVB must be rapidly identified. Unstable temporary pacing with high capture thresholds represents a life-threatening condition requiring urgent permanent pacemaker implantation. HEMS plays a critical role in ensuring timely access to definitive cardiac care in geographically isolated regions.

## 1. Introduction

Syncope, defined as a transient loss of consciousness due to global cerebral hypoperfusion, is a frequent but challenging presentation in emergency medicine, especially among older adults living in remote regions. Acute cardiovascular conditions remain a major cause of emergency hospitalization and are associated with significant morbidity and mortality in older adults [1]. Although many syncopal episodes are benign, a significant proportion are caused by serious cardiovascular conditions, including high-grade conduction disturbances such as complete atrioventricular block (CAVB), which carries a substantial risk of sudden cardiac arrest [2].

In elderly patients, syncope may be accompanied by tonic–clonic movements secondary to profound cerebral hypoperfusion, mimicking epileptic seizures and potentially delaying recognition of an underlying cardiac cause [3,4,5]. Distinguishing convulsive syncope from primary seizure disorders is critical, as misdiagnosis can result in inappropriate treatment and missed opportunities for lifesaving cardiac interventions [3,4,5].

Geographical isolation further complicates management, as access to specialized cardiac services and permanent pacing may be limited or delayed. In this context, helicopter emergency medical services (HEMS) constitute a vital extension of the emergency care system, providing advanced prehospital assessment, stabilization, and rapid transport to tertiary centers.

In Romania, the SMURD Galați air base covers a wide rural area with significant logistical barriers to ground medical transport. In 2024, the Galați HEMS unit conducted approximately 440 missions, including 290 involving hemodynamically unstable patients, 200 with acute cardiovascular pathology, and 80 adults aged 65 years or older.

We report the case of a geriatric patient with CAVB and convulsive-syncope retrieved directly by helicopter from a remote area, whose unstable temporary pacing required urgent aeromedical transfer for permanent pacemaker implantation.

## 2. Case Presentation

A 70-year-old man residing in a remote rural area experienced a sudden tonic–clonic episode during sleep, followed by loss of consciousness. Witnesses reported generalized motor activity lasting several seconds, with spontaneous recovery of consciousness but subsequent recurrent syncopal episodes.

Due to difficult terrain and the long distance to the nearest hospital, the SMURD Galați HEMS team was dispatched. The electrocardiogram performed during the emergency evaluation revealed a complete atrioventricular block with severe bradycardia, explaining the patient’s recurrent episodes of convulsive syncope (Figure 1).

The past medical history was significant for hypertension; no prior conduction abnormalities or structural heart disease had been documented. There was no previous history of syncope, seizures, or diagnosed neurological disorders. At the time of presentation, the patient was not receiving any medications known to impair atrioventricular conduction, such as beta-blockers, non-dihydropyridine calcium channel blockers, digoxin, or antiarrhythmic agents.

### 2.1. Air Medical Initial Assessment

On arrival, the patient was confused but arousable, pale, and hypotensive, with a blood pressure of 90/60 mmHg. The heart rate was approximately 30–35 beats per minute, consistent with a slow ventricular escape rhythm. Oxygen saturation ranged between 91% and 94% on room air, and respiratory examination was unremarkable. Cardiac auscultation revealed a bradycardic rhythm with systolic murmurs. During assessment, the patient experienced recurrent brief syncopal episodes. Electrocardiography performed on scene confirmed complete atrioventricular block with a slow junctional or ventricular escape rhythm.

### 2.2. Temporary Pacing and Aeromedical Transport

Transvenous temporary pacing was established under continuous ECG monitoring. Despite appropriate lead positioning, capture thresholds progressively increased, requiring pacing outputs up to approximately 80 mV, with intermittent loss of capture.

Given the persistent hemodynamic instability, high and fluctuating pacing thresholds, intermittent loss of capture, and the need for urgent permanent pacemaker implantation, the patient was transported by helicopter to a tertiary interventional cardiology center.

During flight, the patient remained under continuous monitoring, and pacing parameters were repeatedly adjusted in response to intermittent loss of capture and episodes of severe bradycardia.

### 2.3. Hospital Course

Following stabilization, additional investigations were performed to assess the etiology of the complete atrioventricular block. Acute coronary syndrome was considered and ruled out based on the absence of chest pain, lack of ischemic electrocardiographic changes, and normal cardiac biomarkers.

No reversible causes such as electrolyte imbalances or drug-induced conduction disturbances were identified. In the absence of evidence for acute ischemia, a degenerative etiology of the conduction system was considered the most likely cause.

Upon arrival at the tertiary center, the patient experienced additional syncopal episodes before undergoing emergent implantation of a single-chamber VVI permanent pacemaker. A single-chamber VVI pacemaker was selected due to the urgent clinical context, the patient’s hemodynamic instability, and the need for rapid and reliable ventricular pacing. Although dual-chamber pacing may offer physiological advantages, a simpler system was considered more appropriate in this acute setting to minimize procedural time and ensure immediate stabilization.

### 2.4. Post-Procedural Outcome

Twenty-four hours after implantation, device interrogation showed a capture threshold of 0.5 V at 0.4 ms, a lead impedance of 1034 ohms, and VVI pacing at 60 bpm. Echocardiography demonstrated preserved left ventricular systolic function, no pericardial effusion, and no significant valvular disease, with no evidence of severe aortic stenosis or clinically relevant mitral regurgitation.

The patient had no recurrent syncopal or seizure-like episodes and was discharged in stable condition with scheduled cardiology follow-up. At discharge, he was prescribed standard post-implantation care.

## 3. Discussion

This case underscores several key aspects of managing life-threatening bradyarrhythmias in elderly patients, particularly those residing in remote geographic regions with delayed access to advanced cardiac care.

### 3.1. Convulsive Syncope and Diagnostic Challenges in Older Adults

Convulsive syncope represents a well-recognized diagnostic challenge, particularly in elderly patients, where transient loss of consciousness may be accompanied by tonic–clonic movements due to severe cerebral hypoperfusion. This presentation can closely mimic epileptic seizures, leading to potential misdiagnosis and inappropriate treatment. In older adults, age-related autonomic dysfunction, reduced cerebral autoregulation, and a higher burden of comorbidities further complicate clinical assessment.

Failure to recognize a cardiac etiology may result in delayed diagnosis and missed opportunities for life-saving interventions, including cardiac pacing. This case highlights the importance of early electrocardiographic evaluation in any elderly patient presenting with transient loss of consciousness associated with seizure-like activity, as prompt identification of conduction disorders such as complete atrioventricular block is critical for appropriate management.

### 3.2. Complete Atrioventricular Block as a Critical Conduction Disorder

Complete atrioventricular block is associated with significant morbidity and mortality, particularly when presenting with syncope [6,7]. Elderly patients are at increased risk due to degenerative changes in the cardiac conduction system, including fibrosis and ischemia.

Temporary pacing remains the standard initial stabilization strategy; however, rapidly increasing capture thresholds, as observed in this case, significantly increase the risk of pacing failure and impending asystole [8,9]. Such instability reflects a critical clinical situation requiring urgent permanent pacemaker implantation.

Delays in definitive pacing have been associated with recurrent syncope, increased risk of traumatic injury, prolonged cerebral hypoperfusion, and higher short-term mortality. Therefore, early recognition and expedited transfer to a center capable of permanent pacemaker implantation are essential.

### 3.3. Etiology of Complete Atrioventricular Block in Elderly Patients

Degenerative fibrosis of the cardiac conduction system represents the most common cause of complete atrioventricular block in elderly patients. However, reversible causes must always be considered and systematically excluded, particularly acute coronary syndrome, electrolyte disturbances, and the effects of cardiotropic medications.

In the present case, the absence of ischemic symptoms, normal cardiac biomarkers, and lack of exposure to atrioventricular node-blocking drugs supported a degenerative etiology. Careful evaluation of these factors is essential, as certain causes of conduction block may be reversible and do not necessarily require permanent pacing. A schematic overview of the main etiological mechanisms and management approach to cardiac syncope in elderly patients is presented in Figure 2.

### 3.4. Air Medical Transport as a Determinant of Timely Intervention

Timely access to permanent pacing is strongly associated with improved survival in patients with unstable complete atrioventricular block [6,10]. Remote geographic areas frequently experience prolonged transport times, limited ground accessibility, and restricted availability of advanced cardiac support.

HEMS provides several advantages in this context, including a significant reduction in time to tertiary cardiac care, the ability to maintain continuous electrocardiographic and hemodynamic monitoring, and the possibility of performing advanced interventions during transport, such as repeated adjustments of pacing parameters. These factors contribute to minimizing delays and reducing the risks associated with unstable transport conditions [10,11].

In this case, continuous monitoring and rapid adjustment of pacing parameters during aeromedical transfer played a critical role in preventing clinical deterioration.

### 3.5. Coordinated Care Between Prehospital and In-Hospital Teams

Optimal outcomes in critical cardiovascular emergencies depend on effective coordination between prehospital emergency teams, emergency departments, and interventional cardiology units [12]. Rapid identification of complete atrioventricular block, timely initiation of temporary pacing, and efficient communication with the receiving cardiology team enabled prompt definitive treatment in this case.

Such integrated care pathways significantly improve time to diagnosis, time to intervention, and overall patient outcomes, particularly in elderly populations.

### 3.6. Challenges Specific to Elderly Patients Living in Isolated Regions

Geriatric patients living in remote areas face multiple vulnerabilities that may negatively influence outcomes. These include delayed presentation due to limited access to medical services, reduced ability to recognize early warning symptoms, a higher likelihood of living alone or being discovered late after an event, and the absence of nearby facilities capable of providing advanced cardiac care, including pacing. Additionally, logistical barriers may significantly delay transport to tertiary centers, further increasing the risk of adverse events.

This case underscores the critical importance of regional air medical networks in delivering equitable emergency cardiac care to older adults regardless of geographic location. Following transfer to a tertiary cardiac center, the patient underwent implantation of a permanent pacemaker (Figure 3), resulting in stabilization of the cardiac rhythm.

### 3.7. Limitations

This report has several limitations. As a single-case report derived from a single-center experience, the findings have limited generalizability and transferability to other clinical settings, as previously highlighted in the literature [13]. Additionally, detailed long-term follow-up data were not available at the time of writing. Despite these limitations, the case provides valuable insights into the diagnostic and logistical challenges associated with managing life-threatening bradyarrhythmias in geographically isolated regions.

## 4. Conclusions

Convulsive-syncopal episodes in elderly patients may reflect severe cerebral hypoperfusion due to cardiac conduction disorders such as complete atrioventricular block rather than primary neurological disease. Unstable temporary pacing with high capture thresholds represents a high-risk condition requiring urgent permanent pacemaker implantation. Air medical transport plays a critical role in enabling rapid access to definitive cardiac care in remote areas. Integrated coordination between prehospital and in-hospital teams is essential for improving outcomes in geriatric cardiovascular emergencies.

## Figures and Tables

**Figure 1:**
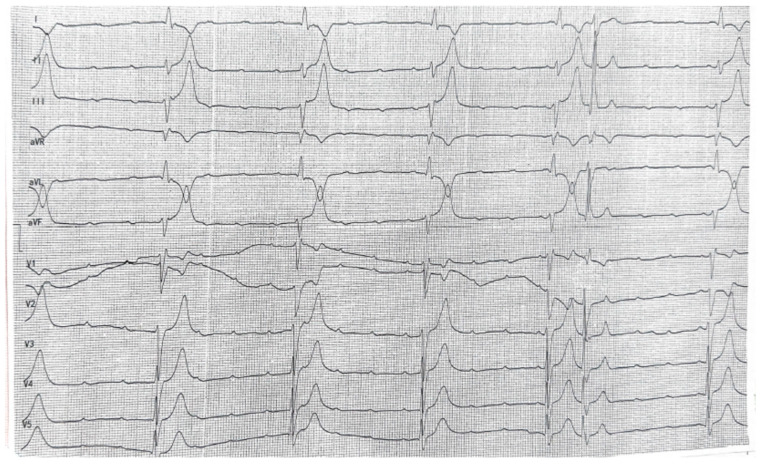
Twelve-lead electrocardiogram recorded during prehospital assessment showing complete atrioventricular block with a slow ventricular escape rhythm.

**Figure 2:**
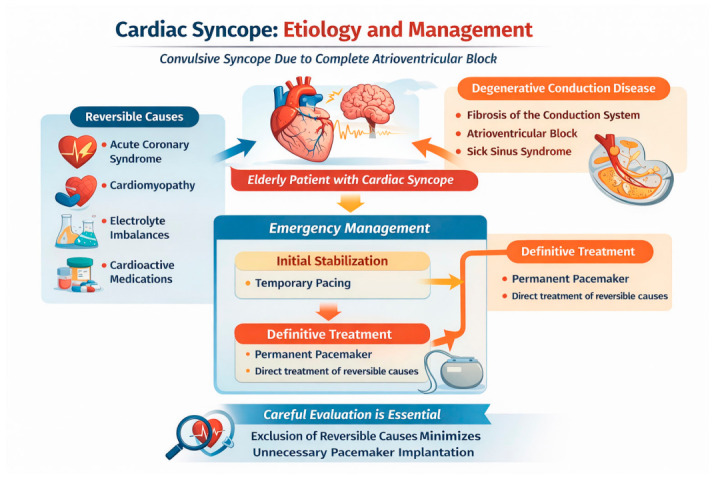
Overview of cardiac syncope in elderly patients, highlighting the main etiological categories and management approach. Reversible causes such as acute coronary syndrome, electrolyte disturbances, and cardiotropic medications must be systematically excluded. Degenerative conduction system disease represents the most common underlying mechanism in older adults. Initial management includes hemodynamic stabilization and temporary pacing, followed by definitive treatment, most commonly permanent pacemaker implantation.

**Figure 3:**
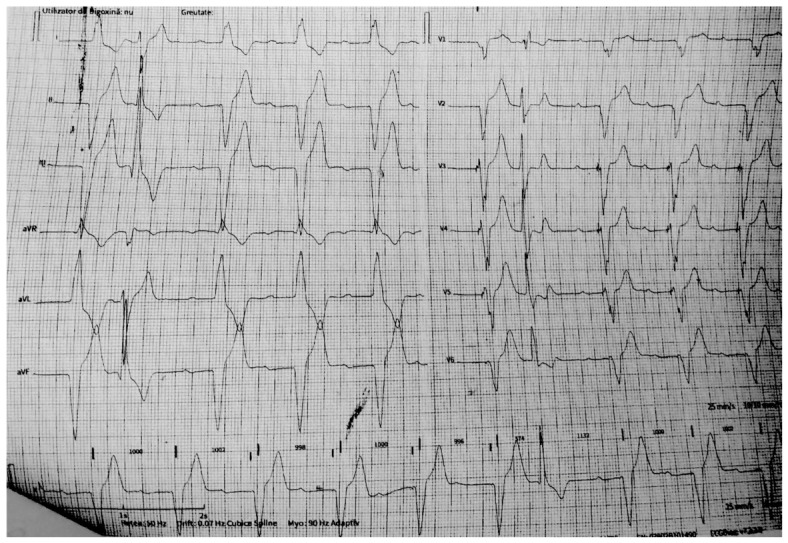
Post-implant electrocardiogram showing effective ventricular pacing following permanent VVI pacemaker implantation.

## Data Availability

Data sharing is not applicable to this article because no new datasets were generated or analyzed beyond the clinical information included in this case report.
